# Quantum Synchronization via Active–Passive Decomposition Configuration: An Open Quantum-System Study

**DOI:** 10.3390/e27040432

**Published:** 2025-04-16

**Authors:** Nan Yang, Ting Yu

**Affiliations:** Center for Quantum Science and Engineering, and Department of Physics, Stevens Institute of Technology, Hoboken, NJ 07030, USA; tyu1@stevens.edu

**Keywords:** quantum synchronization, chaos, optomechanical system

## Abstract

In this paper, we study the synchronization of dissipative quantum harmonic oscillators in the framework of a quantum open system via the active–passive decomposition (APD) configuration. We show that two or more quantum systems may be synchronized when the quantum systems of interest are embedded in dissipative environments and influenced by a common classical system. Such a classical system is typically termed a controller, which (1) can drive quantum systems to cross different regimes (e.g., from periodic to chaotic motions) and (2) constructs the so-called active–passive decomposition configuration, such that all the quantum objects under consideration may be synchronized. The main finding of this paper is that we demonstrate that the complete synchronizations measured using the standard quantum deviation may be achieved for both stable regimes (quantum limit circles) and unstable regimes (quantum chaotic motions). As an example, we numerically show in an optomechanical setup that complete synchronization can be realized in quantum mechanical resonators.

## 1. Introduction

The history of synchronization can be traced back to the Dutch scientist C. Huygens, who first recorded the synchronization behaviors of two suspended pendulums [1]. He also accurately understood the physics behind the phenomena: the oscillators may adjust their rhythms to reach a consistent state due to weak interactions. Since then, synchronization problems have been recognized as a universal phenomenon in nature, ranging from bursting neurons, fireflies, and chemical reactions to human activities, seasonal migrations, and solar systems, as well as the references therein [2,3]. In the domain of classical physics, for example, the synchronization of periodic oscillators may be studied based on the Adler equation [4] and the Kuramoto model [5]. In addition, chaotic synchronization has also been extensively studied by many different methods [6,7,8,9,10,11,12,13]. Among them, the so-called active–passive decomposition (APD) configuration [7,8] provides a general method for the complete synchronization of chaotic systems.

The synchronization problems in quantum systems are also of great interest due to their practical applications and close connections with the fundamental aspects of quantum physics, such as the transition from quantum to classical domains. The related works have been reported in various quantum systems, including atomic systems [14,15,16,17], qubits [18,19,20,21], ions [22], spin models [23,24,25,26,27,28,29], lattices and dimers [30,31,32], Van der Pol oscillators [33,34,35,36,37,38,39], quantum harmonic oscillators [40,41,42,43,44,45,46,47,48,49,50,51], and optomechanical systems [52,53,54,55,56,57]. Quantum synchronization in non-Markovian environments was studied in ref. [58]. It has been experimentally demonstrated that high-frequency resonators may potentially enable quantum synchronization [46,59,60,61,62,63,64]. More applications of quantum synchronization in a quantum network and key distribution may be found in [65,66]. Despite remarkable progress, many interesting problems involving quantum open systems still need to be carefully studied. Particularly, how to synchronize quantum systems in unstable regimes is a long-standing problem.

The purpose of this paper is to study the synchronization of two quantum harmonic oscillators in the quantum open system framework. We demonstrate that quantum synchronization may be achieved for dissipative quantum systems in not only periodic cases for which limit circles are displayed but also the more complex unstable regimes where chaotic dynamics are involved.

For our quantum harmonic oscillator model, it is convenient to use the standard deviations $\sigma_{x}$, $\sigma_{p}$ of the position and momentum operators $\hat{x}$, $\hat{p}$ to measure the degree of quantum synchronization. As shown in this paper, the given quantum synchronization criterion allows us to analytically demonstrate the synchronization between two quantum harmonic oscillators in two separate dissipative environments. Our approach uses the active–passive decomposition (APD) configuration [7,8] to show that the participating classical system can enable the quantum synchronization by constructing an APD configuration and driving the quantum systems into a desired type of motion (e.g., from period to chaos). For the continuous-variable systems considered in this paper, we can reconstruct classical trajectories from quantum systems [67]. Finally, we also numerically show in an experimentally accessible optomechanical setup [68,69,70,71,72,73,74,75,76,77,78,79,80,81,82,83,84,85,86,87,88,89,90,91,92,93,94] that desirable synchronization can be accomplished.

This paper is organized as follows. In Section 2, we introduce the criterion of quantum synchronization for our continuous-variable systems. In Section 3, we discuss the realization of quantum synchronizations in several interesting scenarios. Then, in Section 4, we apply our quantum synchronization approach to an optomechanical setup; our numerical simulations show that the quantum synchronization is achieved in two quantum mechanical resonators in both periodic and chaotic regimes. Finally, we summarize and conclude this paper in Section 5.

## 2. Criteria for Quantum Synchronization

In classical dynamics, the synchronization of two oscillators can be measured using the relative relationships between their trajectories; e.g., complete synchronization refers to approaching identical trajectories. Quantum systems, by default, would not allow us to use concepts like “classical trajectories” directly [95]. In quantum domains, various definitions for synchronization have been proposed, such as the semiclassical trajectory approach [95], the relative phase distribution [22], and the Wigner-function approach [45]. Also, it should be noted that a definition based on observables has been provided recently for the synchronization of quantum limit circles [96].

**Figure 1:**
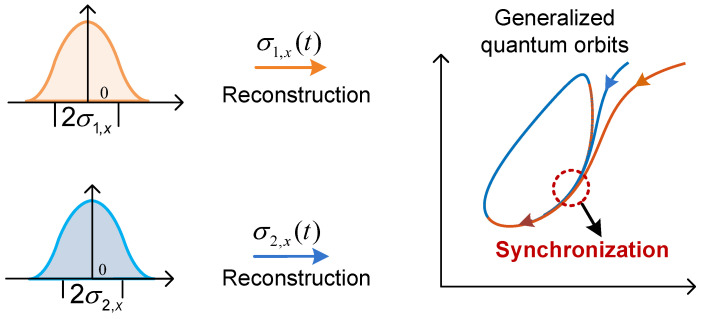
Quantum synchronization between two systems, 1 (organge color) and 2 (blue color), is characterized by the identity of their standard deviations ($\sigma_{1,x},\sigma_{1,p}$) and ($\sigma_{2,x},\sigma_{2,p}$).

For our discussions, the criteria to be used to measure the quantum synchronization for continuous variables are based on the standard deviations [$\sigma_{x}\left(t\right)$, $\sigma_{p}\left(t\right)$] of the position and momentum operators ($\hat{x}$, $\hat{p}$) of the harmonic oscillator systems: $\sigma_{x}=\sqrt{\langle {\hat{x}}^{2}\rangle -{\langle \hat{x}\rangle }^{2}}$ and $\sigma_{p}=\sqrt{\langle {\hat{p}}^{2}\rangle -{\langle \hat{p}\rangle }^{2}}$. Of course, if the quantum system under consideration has other degrees of freedom, such as spins or discrete systems, our definition of synchronization must be modified accordingly [14,15,16,17,18,19,20,21,22,23,24,25,26,27,28,29]. In this way, the synchronization of two quantum systems may be measured by the relative relationships between their standard deviations [$\sigma_{1,x}\left(t\right)$, $\sigma_{1,p}\left(t\right)$] and [$\sigma_{2,x}\left(t\right)$, $\sigma_{2,p}\left(t\right)$] of the quantum position and momentum ($\hat{x}$, $\hat{p}$). Additionally, this definition provides a intuitive framework of understanding of quantum synchronization by employing phase space reconstruction of continuous variables. As shown in Figure 1, the phase space orbits (orange and blue curves) generated from $\sigma_{1,x}\left(t\right)$ and $\sigma_{2,x}\left(t\right)$ converge to each other as the time progresses, indicating that two quantum systems starting from distinct initial conditions achieve a synchronization state though time evolution.

To introduce the detailed definition for quantum synchronization, we first review the definition of complete synchronization in classical systems [2,6].

Complete synchronization in classical contexts refers to the identity of the trajectories of two dynamical systems. Consider two autonomous dynamical systems, ${\dot{y}}_{1}=f\left(y_{1}\right)$ and ${\dot{y}}_{2}=f\left(y_{2}\right)$, where $y_{1}$ and $y_{2}$ are *N*-dimensional variables governed by the function $f:R^{N}\to R^{N}$. Here, $y_{1}$ and $y_{2}$ are called complete synchronization if and only if their difference $\lim_{t\to \infty }∥y_{1}\left(t\right)-y_{2}\left(t\right)∥=0$.

In a similar spirit, here, we show that the above definition can be directly extended to quantum systems when the standard deviations are used.

(**Complete synchronization**) Two quantum systems, 1 and 2, are called complete synchronization if their standard deviations [$\sigma_{1,x}\left(t\right)$, $\sigma_{1,p}\left(t\right)$] and [$\sigma_{2,x}\left(t\right)$, $\sigma_{2,p}\left(t\right)$] satisfy the following conditions:

(1) $$\lim_{t\to \infty }∥\sigma_{1,x}\left(t\right)-\sigma_{2,x}\left(t\right)∥=0,\;\;\;\lim_{t\to \infty }∥\sigma_{1,p}\left(t\right)-\sigma_{2,p}\left(t\right)∥=0,$$

where [$\sigma_{1,x}\left(t\right)$, $\sigma_{1,p}\left(t\right)$] and [$\sigma_{2,x}\left(t\right)$, $\sigma_{2,p}\left(t\right)$] represent the standard deviations of the two quantum systems, 1 and 2.

In the next section, we use these criteria to discuss some physically interesting examples that can display the realization of quantum synchronizations.

## 3. Synchronization of Two Quantum Harmonic Oscillators Based on Active–Passive Decomposition Configuration

To begin with, let us notice that, for classical dynamical systems, the so-called complete synchronization may be accomplished in many different ways, such as mutual interactions, common driving forces, and feedback control mechanisms, etc. Interestingly, synchronization has been extended to some important nonlinear systems where chaotic synchronization may be observed. Chaotic synchronization was once believed not to be feasible. This is because chaos is sensitive to initial conditions, while synchronization requires stability. In 1990, Pecora and Carroll proposed a drive–response (DR) method that successfully synchronized two identical chaotic systems but with different initial conditions: one is called the drive system and the other the response, and they are coupled in a unidirectional way. In this DR method, the response system is supposed to be decomposed into a stable subsystem and an unstable one. Then, the variable of the unstable subsystem is replaced with the corresponding one in the drive system, such that the synchronization error can be eliminated in the stable subsystem. Active–passive decomposition (APD) was derived from the DR model and considered a generalization of the latter. Compared to the DR model, the APD model is not restricted to decomposable chaotic systems, enabling more comprehensive applications in engineering. Especially for n-body quantum systems, the APD model provides a convenient way to construct a general synchronization strategy, where the quantum measurement problem may not be evolved, but quantum synchronization can still be achieved. To conclude, among several useful methods in realizing chaotic synchronization, we find the active–passive decomposition (APD) configuration particularly convenient for our discussions.

To put our discussions into perspective, let us first briefly review the basic idea of active–passive decomposition (APD) configuration. In the APD model, two chaotic subsystems to be synchronized can be written as non-autonomous forms [7]:

(2) $${\dot{z}}_{1}=f\left[z_{1},s\left(t\right)\right],\;{\dot{z}}_{2}=f\left[z_{2},s\left(t\right)\right],$$

where the dynamics of both $z_{1}$ and $z_{2}$ are ruled by the function f, and s(t) is the common external signal governed by the autonomous function $\dot{s}\left(t\right)=h\left[s\left(t\right)\right]$. Here, the synchronization differences e are defined as $e=z_{1}-z_{2}$, and their dynamics are ruled by [7]

(3) $$\dot{e}=f\left[z_{1},s\left(t\right)\right]-f\left[z_{1}-e,s\left(t\right)\right].$$

The APD model constructs a configuration in which Equation (3) is asymptotically stable at the zero point e=0. Thus, the synchronization differences e go to zero as the time increases, and complete synchronization occurs for two chaotic dynamical systems, $z_{1}$ and $z_{2}$.

**Figure 2:**
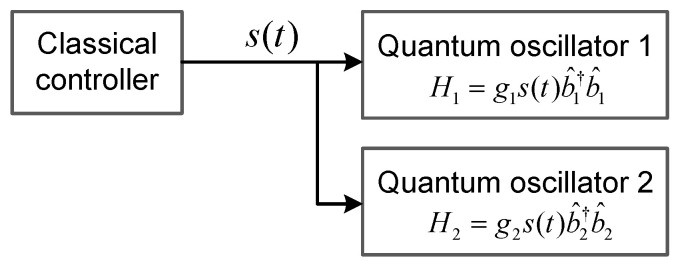
Schematic diagram for the synchronization of two quantum harmonic oscillators, ${\hat{b}}_{1}$ and ${\hat{b}}_{2}$.

As is shown below, the active–passive decomposition (APD) configuration [7,8] may also be applied to the synchronization of quantum systems. As shown in Figure 2, our quantum model consists of two separate quantum harmonic oscillators (${\hat{b}}_{1}$ and ${\hat{b}}_{2}$) in different heat baths and a classical controller that produces a common frequency modulation signal, s(t), acting on ${\hat{b}}_{1}$ and ${\hat{b}}_{2}$ simultaneously. Here, the effective Hamiltonian of the two harmonic oscillators is given by

(4) $$H=\hbar \left[\Omega_{1}+g_{1}s\left(t\right)\right]{\hat{b}}_{1}^{†}{\hat{b}}_{1}+\hbar \left[\Omega_{2}+g_{2}s\left(t\right)\right]{\hat{b}}_{2}^{†}{\hat{b}}_{2},$$

where $\Omega_{j}$ is the resonant frequency of the j−th quantum harmonic oscillator, ${\hat{b}}_{j}$. The term $g_{j}s\left(t\right)$ refers to the frequency shift of ${\hat{b}}_{j}$ brought via the classical input s(t), and $g_{j}$ is the coupling strength. In this setting, the quantum dynamics are modulated by the classical controller, which can be driven from periodic to chaotic regimes by adjusting the classical input, s(t). In what follows, we first derive the equations of motions for the second-order terms of the quantum harmonic oscillators (${\hat{b}}_{1}$ and ${\hat{b}}_{2}$), which determine the dynamics of the corresponding standard deviations [$\sigma_{1,x}$, $\sigma_{1,p}$] and [$\sigma_{2,x}$, $\sigma_{2,p}$]. Then, we demonstrate that the quantum synchronization of two harmonic oscillators is achievable and stable in the APD configuration, and the stability is robust for both periodic and chaotic motions. Below, we provide an intuitive physical understanding of the APD configuration of quantum chaotic synchronization. An autonomous chaotic system is known to be sensitive to initial conditions, such as a Lorenz attractor. However, in this paper, quantum chaos is imported from the classical one [Figure 2], which makes the quantum chaotic systems non-autonomous. In fact, the quantum chaotic systems subject to dissipation are stable subsystems that are controlled via the unstable (chaotic) classical system. With this regime, small quantum fluctuations that exist in the systems will not be rapidly amplified in this process. Also, we show in what follows that the initial differences between two quantum chaotic systems will be eliminated due to dissipation.

### 3.1. The Equations of Motions for the Second-Order Terms of the Quantum Harmonic Oscillators ${\hat{b}}_{1}$ and ${\hat{b}}_{2}$

For convenience, we use the shifted quantum harmonic oscillators ${\hat{b}}_{1}$ and ${\hat{b}}_{2}$

(5) $${\hat{b}}_{1}=\beta_{1}+{\hat{\tilde{b}}}_{1},\;\;\;{\hat{b}}_{2}=\beta_{2}+{\hat{\tilde{b}}}_{2},$$

where $\beta_{j}=\langle {\hat{b}}_{j}\rangle$ refers to the classical mean value, and ${\hat{\tilde{b}}}_{j}$ is the quantum fluctuation term of the mechanical mode ${\hat{b}}_{j}$ ($\langle {\hat{\tilde{b}}}_{j}\rangle =0$). Then, the Hamiltonian in terms of ${\hat{\tilde{b}}}_{j}$(j=1,2) is obtained by substituting Equation (5) into Equation (4)

(6) $$H=\hbar \Omega_{1}^{\prime }\left(t\right){\hat{\tilde{b}}}_{1}^{†}{\hat{\tilde{b}}}_{1}+\hbar \Omega_{2}^{\prime }\left(t\right){\hat{\tilde{b}}}_{2}^{†}{\hat{\tilde{b}}}_{2},$$

where $\Omega_{j}^{\prime }\left(t\right)=\Omega_{j}+g_{j}s\left(t\right)$ is the modified resonant frequency of the j−th quantum harmonic oscillator ${\hat{b}}_{j}$ for j=1,2. The dynamics of the two quantum harmonic oscillators in two separate heat baths can be described using the master equation [97,98,99,100,101]; a few approximations, such as Markovian approximation, are applied in this process. With the system Hamiltonian given by Equation (6), the Markovian master equation is given by

(7) $$\begin{array}{cc} \dot{\rho }= & i\left[\rho ,\hbar \Omega_{1}^{\prime }\left(t\right){\hat{\tilde{b}}}_{1}^{†}{\hat{\tilde{b}}}_{1}+\hbar \Omega_{2}^{\prime }\left(t\right){\hat{\tilde{b}}}_{2}^{†}{\hat{\tilde{b}}}_{2}\right]\\ \; & +\sum_{j=1,2}\left[\Gamma_{j}\left[n_{j,\mathrm{th}}\left(t\right)+1\right]\left(2{\hat{\tilde{b}}}_{j}\rho {\hat{\tilde{b}}}_{j}^{†}-{\hat{\tilde{b}}}_{j}^{†}{\hat{\tilde{b}}}_{j}\rho -\rho {\hat{\tilde{b}}}_{j}^{†}{\hat{\tilde{b}}}_{j}\right)\right]\\ \; & +\sum_{j=1,2}\left[\Gamma_{j}n_{j,\mathrm{th}}\left(t\right)\left(2{\hat{\tilde{b}}}_{j}^{†}\rho {\hat{\tilde{b}}}_{j}-{\hat{\tilde{b}}}_{j}{\hat{\tilde{b}}}_{j}^{†}\rho -\rho {\hat{\tilde{b}}}_{j}{\hat{\tilde{b}}}_{j}^{†}\right)\right], \end{array}$$

where $\Gamma_{j}$ is the damping rate of the j−th quantum harmonic oscillator $b_{j}$, and its mean thermal photon (phonon) excitation number $n_{j,\mathrm{th}}\left(t\right)=\exp{\left[\hbar \Omega_{j}^{\prime }\left(t\right)/\kappa_{B}T_{j}-1\right]}^{-1}$ is determined by the temperature, $T_{j}$, and the effective resonant frequency, $\Omega_{j}^{\prime }\left(t\right)$. Here, $\kappa_{B}$ is the Boltzmann constant. From the master equation, we can then obtain the equations of motions for $\langle {\hat{\tilde{b}}}_{1}^{†}{\hat{\tilde{b}}}_{1}\rangle$, $\langle {\hat{\tilde{b}}}_{2}^{†}{\hat{\tilde{b}}}_{2}\rangle$, $\langle {\hat{\tilde{b}}}_{1}^{†}{\hat{\tilde{b}}}_{2}\rangle$, $\langle {\hat{\tilde{b}}}_{1}{\hat{\tilde{b}}}_{2}\rangle$, $\langle {\hat{\tilde{b}}}_{1}^{2}\rangle$, and $\langle {\hat{\tilde{b}}}_{2}^{2}\rangle$ by applying $\langle \dot{\hat{o}}\rangle =\mathrm{Tr}\left(\rho \hat{o}\right)$ for the operator $\hat{o}$,

(8) $$\begin{array}{cc} \frac{d\langle {\hat{\tilde{b}}}_{1}^{†}{\hat{\tilde{b}}}_{1}\rangle }{dt}= & -\Gamma_{1}\langle {\hat{\tilde{b}}}_{1}^{†}{\hat{\tilde{b}}}_{1}\rangle +\Gamma_{1}n_{j,\mathrm{th}}\left[\Omega_{1}^{\prime }\left(t\right)\right],\\ \frac{d\langle {\hat{\tilde{b}}}_{2}^{†}{\hat{\tilde{b}}}_{2}\rangle }{dt}= & -\Gamma_{2}\langle {\hat{\tilde{b}}}_{2}^{†}{\hat{\tilde{b}}}_{2}\rangle +\Gamma_{2}n_{j,\mathrm{th}}\left[\Omega_{2}^{\prime }\left(t\right)\right],\\ \frac{d\langle {\hat{\tilde{b}}}_{1}^{†}{\hat{\tilde{b}}}_{2}\rangle }{dt}= & -i\left[-\Omega_{1}^{\prime }\left(t\right)+\Omega_{2}^{\prime }\left(t\right)\right]\langle {\hat{\tilde{b}}}_{1}^{†}{\hat{\tilde{b}}}_{2}\rangle -\frac{\Gamma_{1}+\Gamma_{2}}{2}\langle {\hat{\tilde{b}}}_{1}^{†}{\hat{\tilde{b}}}_{2}\rangle ,\\ \frac{d\langle {\hat{\tilde{b}}}_{1}{\hat{\tilde{b}}}_{2}\rangle }{dt}= & -i\left[\Omega_{1}^{\prime }\left(t\right)+\Omega_{2}^{\prime }\left(t\right)\right]\langle {\hat{\tilde{b}}}_{1}{\hat{\tilde{b}}}_{2}\rangle -\frac{\Gamma_{1}+\Gamma_{2}}{2}\langle {\hat{\tilde{b}}}_{1}{\hat{\tilde{b}}}_{2}\rangle ,\\ \frac{d\langle {\hat{\tilde{b}}}_{1}^{2}\rangle }{dt}= & \left[-2i\Omega_{1}^{\prime }\left(t\right)-\Gamma_{1}\right]\langle {\hat{\tilde{b}}}_{1}^{2}\rangle ,\\ \frac{d\langle {\hat{\tilde{b}}}_{2}^{2}\rangle }{dt}= & \left[-2i\Omega_{2}^{\prime }\left(t\right)-\Gamma_{2}\right]\langle {\hat{\tilde{b}}}_{2}^{2}\rangle . \end{array}$$

One can easily find that the values of $\langle {\hat{\tilde{b}}}_{1}^{†}{\hat{\tilde{b}}}_{2}\rangle$, $\langle {\hat{\tilde{b}}}_{1}{\hat{\tilde{b}}}_{2}\rangle$, $\langle {\hat{\tilde{b}}}_{1}^{2}\rangle$, and $\langle {\hat{\tilde{b}}}_{2}^{2}\rangle$ decay to zero since they couple to dissipation but are not subject to any driving. Here, for the non-zero terms $\langle {\hat{\tilde{b}}}_{1}^{†}{\hat{\tilde{b}}}_{1}\rangle$ and $\langle {\hat{\tilde{b}}}_{2}^{†}{\hat{\tilde{b}}}_{2}\rangle$, their dynamics are dominated by $n_{1,\mathrm{th}}\left(t\right)$ and $n_{2,\mathrm{th}}\left(t\right)$, respectively, where $n_{1,\mathrm{th}}\left(t\right)$$\left[n_{2,\mathrm{th}}\left(t\right)\right]$ is known as a function of the modified mechanical frequency $\Omega_{1}^{\prime }\left(t\right)$ [$\Omega_{2}^{\prime }\left(t\right)$].

### 3.2. Quantum Synchronization of Dissipative Harmonic Oscillators

Recall the definition of quantum synchronization discussed in Section 2, where the complete synchronization of two quantum harmonic oscillators, ${\hat{b}}_{1}$ and ${\hat{b}}_{2}$, is achieved if their standard deviations [$\sigma_{1,x}\left(t\right)$, $\sigma_{1,p}\left(t\right)$] and [$\sigma_{2,x}\left(t\right)$, $\sigma_{2,p}\left(t\right)$] satisfy the following conditions: $\lim_{t\to \infty }\left[\sigma_{1,x}\left(t\right)-\sigma_{2,x}\left(t\right)\right]=0$ and $\lim_{t\to \infty }\left[\sigma_{1,p}\left(t\right)-\sigma_{2,p}\left(t\right)\right]=0$. Here, $\sigma_{j,x}$ and $\sigma_{j,p}$ take the forms

(9) $$\sigma_{j,x}=\sqrt{\langle {\hat{x}}_{j}^{2}\rangle -{\langle {\hat{x}}_{j}\rangle }^{2}},\;\;\;\;\;\sigma_{j,p}=\sqrt{\langle {\hat{p}}_{j}^{2}\rangle -{\langle {\hat{p}}_{j}\rangle }^{2}},j=1,2.$$

With the application of the relations given in Equation (5), the standard deviations $\sigma_{j,x}$ and $\sigma_{j,p}$ of the quantum harmonic oscillator ${\hat{b}}_{j}$ are rewritten as

(10) σj,x=12+〈b˜^j†b˜^j〉+Re[〈b˜^j2〉],σj,p=12+〈b˜^j†b˜^j〉−Re[〈b˜^j2〉],j=1,2.

It can be seen from Equation (10) that both $\sigma_{j,x}$ and $\sigma_{j,p}$ are functions of $\langle {\hat{\tilde{b}}}_{j}^{†}{\hat{\tilde{b}}}_{j}\rangle$ and $\langle {\hat{\tilde{b}}}_{j}^{2}\rangle$; thus, we have new conditions for quantum complete synchronization,

(11) $$\lim_{t\to \infty }e_{n_{b}}\left(t\right)=0,\;\;\;\;\;\lim_{t\to \infty }e_{b^{2}}\left(t\right)=0,$$

where $e_{n_{b}}=\langle {\hat{\tilde{b}}}_{1}^{†}{\hat{\tilde{b}}}_{1}\rangle -\langle {\hat{\tilde{b}}}_{2}^{†}{\hat{\tilde{b}}}_{2}\rangle$ and $e_{b^{2}}=\langle {\hat{\tilde{b}}}_{1}^{2}\rangle -\langle {\hat{\tilde{b}}}_{2}^{2}\rangle$ stand for the synchronization differences. Also, the equations of motion for $\langle {\hat{\tilde{b}}}_{j}^{†}{\hat{\tilde{b}}}_{j}\rangle$ and $\langle {\hat{\tilde{b}}}_{j}^{2}\rangle$ can be found in Equation (8)

(12) $$\begin{array}{cc} d\langle {\hat{\tilde{b}}}_{j}^{†}{\hat{\tilde{b}}}_{j}\rangle /dt & =-\Gamma_{j}\langle {\hat{\tilde{b}}}_{j}^{†}{\hat{\tilde{b}}}_{j}\rangle +\Gamma_{j}n_{j,\mathrm{th}}\left[\Omega_{j}^{\prime }\left(t\right)\right],\\ d\langle {\hat{\tilde{b}}}_{j}^{2}\rangle /dt & =-\left[2i\Omega_{j}^{\prime }\left(t\right)+\Gamma_{j}\right]\langle {\hat{\tilde{b}}}_{j}^{2}\rangle ,j=1,2. \end{array}$$

For complete synchronization, note that all the parameters of two quantum harmonic oscillators are required to be identical: $\Gamma_{1}=\Gamma_{2}=\Gamma$, $g_{1}=g_{2}=g$, and $\Omega_{1}=\Omega_{2}=\Omega$. Since the two oscillators also share the same input, s(t) (a necessary condition for APD configuration), we have $\Omega_{1}^{\prime }\left(t\right)=\Omega_{2}^{\prime }\left(t\right)=\Omega^{\prime }\left(t\right)$. Then, one can easily obtain the equations of motion for the synchronization differences from Equation (12)

(13) $$\begin{array}{cc} {\dot{e}}_{n_{b}} & =-\Gamma e_{n_{b}},\\ {\dot{e}}_{b^{2}} & =-\left[2i\Omega^{\prime }\left(t\right)+\Gamma \right]e_{b^{2}}. \end{array}$$

To check whether the quantum synchronization conditions provided by Equation (11) can be satisfied, we analytically solve Equation (13) and obtain the following solutions: $e_{n_{b}}\left(t\right)=\exp\left(-\Gamma t\right)e_{n_{b}}\left(0\right)$ and $e_{b^{2}}\left(t\right)=\exp\left(-2\Gamma t\right)\exp\int_{0}^{t}\left[-2i\Omega^{\prime }\left(t^{\prime }\right)\right]dt^{\prime }e_{b^{2}}\left(0\right)$. We find that both of the synchronization differences, $e_{n_{b}}$ and $e_{b^{2}}$, converge to zero as t→∞ when Γ>0. It thus can be concluded that the quantum synchronization led by this approach is asymptotically stable when the quantum harmonic oscillators are subject to dissipation.

The above discussion has demonstrated that the complete synchronization criterion of two quantum harmonic oscillators given in Section 2 can be achieved using the APD configuration method. The stability of the complete quantum synchronization induced via the environment has implied that it can also be realized in chaotic regimes with the same mechanism to be discussed in the next section.

## 4. A Quantum Synchronization Model: Periodic and Chaotic Motions

In this section, we study the implementation of the above quantum synchronization model with an experimentally accessible optomechanical setup. We numerically show that complete synchronization can be achieved in this mode that consists of two quantum mechanical resonators. Interestingly, this simple model actually allows us to show that quantum synchronization is robust in either periodic or chaotic regimes [102,103,104,105,106,107,108,109,110,111,112,113,114,115,116,117,118,119,120,121,122,123,124,125,126,127,128,129,130].

**Figure 3:**
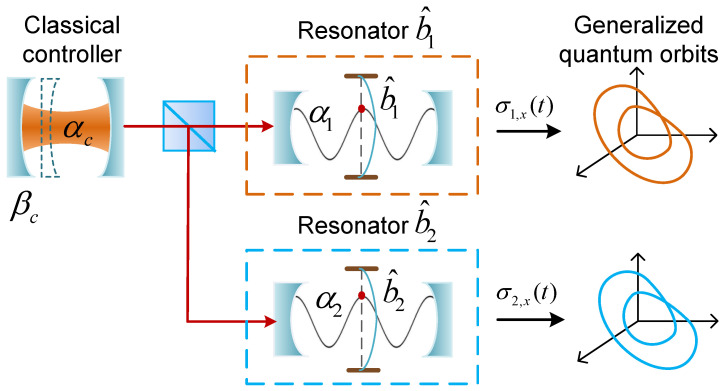
An optomechanical setup for the quantum synchronization of two mechanical resonators, ${\hat{b}}_{1}$ and ${\hat{b}}_{2}$, in the framework of APD configuration.

As shown in Figure 3, the setup consists of three components: an optomechanical system (left side) and two quadratic-coupling optomechanical systems (right side). The two quantum mechanical resonators (${\hat{b}}_{1}$ and ${\hat{b}}_{2}$) to be synchronized are distributed in the two separated quadratic-coupling optomechanical systems (right side), respectively. The left-side optomechanical system ($\alpha_{c}$, $\beta_{c}$) is strongly driven and thus can be treated classically. Its outputs are a classical field that acts as the inputs of the cavity fields ($\alpha_{1}$ and $\alpha_{2}$). The quantum mechanical modes (${\hat{b}}_{1}$ and ${\hat{b}}_{2}$) are modulated through the left-side optomechanical system ($\alpha_{c}$, $\beta_{c}$) via quadratic coupling with the cavity fields ($\alpha_{1}$ and $\alpha_{2}$). These form an APD configuration that will lead to the complete synchronization of the two quantum mechanical resonators (${\hat{b}}_{1}$ and ${\hat{b}}_{2}$). Here, the Hamiltonian of the total system reads

(14) $$\begin{array}{cc} H_{\mathrm{total}}= & \Delta_{c}{\hat{a}}_{c}^{†}{\hat{a}}_{c}+\Omega_{c}{\hat{b}}_{c}^{†}{\hat{b}}_{c}+g_{c}{\hat{a}}_{c}^{†}{\hat{a}}_{c}\left({\hat{b}}_{c}^{†}+{\hat{b}}_{c}\right)+\varepsilon_{c}\left({\hat{a}}_{c}^{†}+{\hat{a}}_{c}\right)\\ \; & +\Delta_{1}{\hat{a}}_{1}^{†}{\hat{a}}_{1}+\Delta_{2}{\hat{a}}_{2}^{†}{\hat{a}}_{2}+\Omega_{2}{\hat{b}}_{1}^{†}{\hat{b}}_{1}+\Omega_{2}{\hat{b}}_{2}^{†}{\hat{b}}_{2}\\ \; & +g_{1}{\hat{a}}_{1}^{†}{\hat{a}}_{1}{\hat{b}}_{1}^{†}{\hat{b}}_{1}+g_{2}{\hat{a}}_{2}^{†}{\hat{a}}_{2}{\hat{b}}_{2}^{†}{\hat{b}}_{2}, \end{array}$$

where $\Delta_{k}=\omega_{k}-\omega_{k,d}$ is the detuning between the resonant frequency $\omega_{k}$ and the external driving $\omega_{k,d}$ of the cavity mode ${\hat{a}}_{k}$ for k=1,2,c, and its driving strength is denoted as $\varepsilon_{k}$. Here, the resonant frequency and the damping rate of the mechanical mode ${\hat{b}}_{k}$ are $\Omega_{k}$ and $\Gamma_{k}$, respectively; meanwhile, $g_{k}$ is the optomechanical coupling strength between the cavity mode, ${\hat{a}}_{k}$, and the mechanical resonator, ${\hat{b}}_{k}$.

Here, the equations of motion of each cavity (mechanical) mode are described by the Langevin equations

(15) $$\begin{array}{cc} {\dot{\hat{a}}}_{c} & =-i\Delta_{c}{\hat{a}}_{c}-ig_{c}{\hat{a}}_{c}\left({\hat{b}}_{c}^{†}+{\hat{b}}_{c}\right)-\frac{\gamma_{c}}{2}{\hat{a}}_{c}+\varepsilon_{c}-\sqrt{\gamma_{c}}\;{\hat{a}}_{c,\mathrm{in}},\;\\ {\dot{\hat{b}}}_{c} & =-i\Omega_{c}{\hat{b}}_{c}-ig_{c}{\hat{a}}_{c}^{†}{\hat{a}}_{c}-\frac{\Gamma_{\;c}}{2}{\hat{b}}_{c}-\sqrt{\Gamma_{\;c}}\;{\hat{b}}_{c,\mathrm{in}},\;\\ {\dot{\hat{a}}}_{1} & =-i\Delta_{1}{\hat{a}}_{1}-\frac{\gamma_{1}}{2}{\hat{a}}_{1}-ig_{1}{\hat{a}}_{1}{\hat{b}}_{1}^{†}{\hat{b}}_{1}-\sqrt{\gamma_{1}\gamma_{c}}\;{\hat{a}}_{c}-\sqrt{\gamma_{1}}{\hat{a}}_{1,\mathrm{in}},\;\\ {\dot{\hat{a}}}_{2} & =-i\Delta_{2}{\hat{a}}_{2}-\frac{\gamma_{2}}{2}{\hat{a}}_{2}-ig_{2}{\hat{a}}_{2}{\hat{b}}_{2}^{†}{\hat{b}}_{2}-\sqrt{\gamma_{2}\gamma_{c}}\;{\hat{a}}_{c}-\sqrt{\gamma_{2}}{\hat{a}}_{2,\mathrm{in}},\;\\ {\dot{\hat{b}}}_{1} & =-i\Omega_{1}{\hat{b}}_{1}-\frac{\Gamma_{1}}{2}{\hat{b}}_{1}-ig_{1}{\hat{a}}_{1}^{†}{\hat{a}}_{1}{\hat{b}}_{1}^{†}-\sqrt{\Gamma_{1}}{\hat{b}}_{1,\mathrm{in}},\;\\ {\dot{\hat{b}}}_{2} & =-i\Omega_{2}{\hat{b}}_{2}-\frac{\Gamma_{2}}{2}{\hat{b}}_{2}-ig_{2}{\hat{a}}_{2}^{†}{\hat{a}}_{2}{\hat{b}}_{2}^{†}-\sqrt{\Gamma_{2}}{\hat{b}}_{2,\mathrm{in}},\; \end{array}$$

where $\gamma_{k}$ and ${\hat{a}}_{k,\mathrm{in}}$ are the damping rate and the input of the optical cavity ${\hat{a}}_{k}$, and ${\hat{b}}_{k,\mathrm{in}}$ and $\Gamma_{\;k}$ are the input and the damping rate of the mechanical mode ${\hat{b}}_{k}$ for k=1,2,c.

In this setup, the optomechanical resonator (${\hat{a}}_{c}$, ${\hat{b}}_{c}$) and the cavity modes (${\hat{a}}_{1}$ and ${\hat{a}}_{2}$) can be considered classically. By replacing the quantum operators with their classical averages in Equation (15): $\alpha_{1}=\langle {\hat{a}}_{1}\rangle$, $\alpha_{2}=\langle {\hat{a}}_{2}\rangle$, $\alpha_{c}=\langle {\hat{a}}_{c}\rangle$, and $\beta_{c}=\langle {\hat{b}}_{c}\rangle$, we can then obtain the equations of motions for the classical parts

(16) $$\begin{array}{cc} {\dot{\alpha }}_{c} & =-i\Delta_{c}\alpha_{c}-\frac{\gamma_{c}}{2}\alpha_{c}-ig_{c}\alpha_{c}\left(\beta_{c}+\beta_{c}^{*}\right)+\varepsilon_{c},\\ {\dot{\beta }}_{c} & =\left(-i\Omega_{c}-\frac{\Gamma_{c}}{2}\right)\beta_{c}-ig_{c}{\left|\alpha_{c}\right|}^{2},\\ {\dot{\alpha }}_{1} & =-i\Delta_{1}\alpha_{1}-\frac{\gamma_{1}}{2}\alpha_{1}-\sqrt{\gamma_{1}\gamma_{c}}\alpha_{c}+\varepsilon_{1},\\ {\dot{\alpha }}_{2} & =-i\Delta_{2}\alpha_{2}-\frac{\gamma_{2}}{2}\alpha_{2}-\sqrt{\gamma_{2}\gamma_{c}}\alpha_{c}+\varepsilon_{2}. \end{array}$$

Note that the cavity modes $\alpha_{1}$ and $\alpha_{2}$ are not affected by the mechanical resonators ${\hat{b}}_{1}$ and ${\hat{b}}_{2}$. This is because the latter does not contain driving terms; as such, the classical averages $\beta_{1}$ and $\beta_{2}$ converge to zero as the time *t* increases.

Now, we focus on the dynamical evolution of the quantum mechanical resonator ${\hat{b}}_{1}$ and ${\hat{b}}_{2}$. Since the optomechanical resonator (${\hat{a}}_{c}$, ${\hat{b}}_{c}$) and the cavity modes (${\hat{a}}_{1}$ and ${\hat{a}}_{2}$) treated classically are omitted in the total Hamiltonian [Equation (14)], we have the effective system Hamiltonian,

(17) $${\tilde{H}}_{\mathrm{eff}}=\hbar \Omega_{1}^{\prime }\left(t\right){\hat{b}}_{1}^{†}{\hat{b}}_{1}+\hbar \Omega_{2}^{\prime }\left(t\right){\hat{b}}_{2}^{†}{\hat{b}}_{2},$$

where $\Omega_{j}^{\prime }=\Omega_{j}+g_{j}{\left|\alpha_{j}\right|}^{2}$ is the modified mechanical frequency due to the optomechanical coupling with the classical optical field $\alpha_{j}$ for j=1,2. Here, the cavity mode $\alpha_{1}$ ($\alpha_{2}$) links both the classical optomechanical system ($\alpha_{c}$, $\beta_{c}$) and the quantum mechanical mode ${\hat{b}}_{1}$ (${\hat{b}}_{2}$) together. The chaos generated via the optomechanical system ($\alpha_{c}$, $\beta_{c}$) is thus transferred into the quantum mechanical resonators ${\hat{b}}_{1}$ and ${\hat{b}}_{2}$.

Recall that the dynamics of the quantum mechanical mode ${\hat{b}}_{1}$ (${\hat{b}}_{2}$) are described by the temporal evolution of its standard deviations ($\sigma_{1,x}$, $\sigma_{1,p}$) [($\sigma_{2,x}$, $\sigma_{2,p}$)], which are given in Equation (10). Specifically, in this quadratic-coupling optomechanical setting, the mean value of the mechanical resonator ${\hat{b}}_{1}$ (${\hat{b}}_{2}$) is always zero: $\langle {\hat{b}}_{1}\rangle =0$ ($\langle {\hat{b}}_{2}\rangle =0$). Thus, the standard deviations ($\sigma_{j,x}$, $\sigma_{j,p}$) for the quantum mechanical mode ${\hat{b}}_{1}$ (${\hat{b}}_{2}$) can be rewritten as

(18) σj,x=12+〈b^j†b^j〉+Re[〈b^j2〉],σj,p=12+〈b^j†b^j〉−Re[〈b^j2〉],j=1,2,

where the equations of motions for $\langle {\hat{b}}_{1}^{†}{\hat{b}}_{1}\rangle$, $\langle {\hat{b}}_{2}^{†}{\hat{b}}_{2}\rangle$, $\langle {\hat{b}}_{1}^{†}{\hat{b}}_{2}\rangle$, $\langle {\hat{b}}_{1}{\hat{b}}_{2}\rangle$, $\langle {\hat{b}}_{1}^{2}\rangle$, and $\langle {\hat{b}}_{2}^{2}\rangle$ are given by

(19) $$\begin{array}{cc} \frac{d\langle {\hat{b}}_{1}^{†}{\hat{b}}_{1}\rangle }{dt}= & -\Gamma_{1}\langle {\hat{b}}_{1}^{†}{\hat{b}}_{1}\rangle +\Gamma_{1}n_{1,\mathrm{th}}\left[\Omega_{1}^{\prime }\left(t\right)\right],\\ \frac{d\langle {\hat{b}}_{2}^{†}{\hat{b}}_{2}\rangle }{dt}= & -\Gamma_{2}\langle {\hat{b}}_{2}^{†}{\hat{b}}_{2}\rangle +\Gamma_{2}n_{2,\mathrm{th}}\left[\Omega_{2}^{\prime }\left(t\right)\right],\\ \frac{d\langle {\hat{b}}_{1}^{†}{\hat{b}}_{2}\rangle }{dt}= & -i\left[-\Omega_{1}^{\prime }\left(t\right)+\Omega_{2}^{\prime }\left(t\right)\right]\langle {\hat{b}}_{1}^{†}{\hat{b}}_{2}\rangle -\frac{\Gamma_{1}+\Gamma_{2}}{2}\langle {\hat{b}}_{1}^{†}{\hat{b}}_{2}\rangle ,\\ \frac{d\langle {\hat{b}}_{1}{\hat{b}}_{2}\rangle }{dt}= & -i\left[\Omega_{1}^{\prime }\left(t\right)+\Omega_{2}^{\prime }\left(t\right)\right]\langle {\hat{b}}_{1}{\hat{b}}_{2}\rangle -\frac{\Gamma_{1}+\Gamma_{2}}{2}\langle {\hat{b}}_{1}{\hat{b}}_{2}\rangle ,\\ \frac{d\langle {\hat{b}}_{1}^{2}\rangle }{dt}= & \left[-2i\Omega_{1}^{\prime }\left(t\right)-\Gamma_{1}\right]\langle {\hat{b}}_{1}^{2}\rangle ,\\ \frac{d\langle {\hat{b}}_{2}^{2}\rangle }{dt}= & \left[-2i\Omega_{2}^{\prime }\left(t\right)-\Gamma_{2}\right]\langle {\hat{b}}_{2}^{2}\rangle . \end{array}$$

One can easily find that the values of $\langle {\hat{\tilde{b}}}_{1}^{†}{\hat{\tilde{b}}}_{2}\rangle$, $\langle {\hat{\tilde{b}}}_{1}{\hat{\tilde{b}}}_{2}\rangle$, $\langle {\hat{\tilde{b}}}_{1}^{2}\rangle$, and $\langle {\hat{\tilde{b}}}_{2}^{2}\rangle$ decay to zero as the time, *t*, increases. For the inhomogeneous equations $\langle {\hat{\tilde{b}}}_{1}^{†}{\hat{\tilde{b}}}_{1}\rangle$ ($\langle {\hat{\tilde{b}}}_{2}^{†}{\hat{\tilde{b}}}_{2}\rangle$), the time evolution of these terms will be sensitive to $n_{1,\mathrm{th}}$ ($n_{2,\mathrm{th}}$), where $n_{j,\mathrm{th}}\left(t\right)=\exp{\left[\hbar \Omega_{j}^{\prime }\left(t\right)/\kappa_{B}T-1\right]}^{-1}$ is the mean thermal phonon number at the temperature, *T*, and the modified resonant frequency $\Omega_{j}^{\prime }\left(t\right)$ in this setting.

Also, since limt→∞Re[〈b^j2〉]=0 (j=1,2), we have the relation $\sigma_{j,x}\left(t\right)=\sigma_{j,p}\left(t\right)$ from Equation (18); the condition for quantum synchronization in this setting is simplified as

(20) $$\lim_{t\to \infty }\left[\sigma_{1,x}\left(t\right)-\sigma_{2,x}\left(t\right)\right]=0.$$

Here, the temporal evolutions of the standard deviations $\sigma_{j,x}$ and $\sigma_{j,p}$ are determined by $\langle {\hat{b}}_{j}^{†}{\hat{b}}_{j}\rangle$, whose equations of motion are governed by

(21) $$\frac{d\langle {\hat{b}}_{j}^{†}{\hat{b}}_{j}\rangle }{dt}=-\Gamma_{j}\langle {\hat{b}}_{j}^{†}{\hat{b}}_{j}\rangle +\Gamma_{j}n_{j,\mathrm{th}}\left[\Omega_{j}^{\prime }\left(t\right)\right],j=1,2.$$

To achieve the complete synchronization, the parameters and the classical inputs of the two quantum mechanical modes, ${\hat{b}}_{1}$ and ${\hat{b}}_{2}$, are assumed to be identical: $\Omega_{1}=\Omega_{2}$, $\Gamma_{1}=\Gamma_{2}$, and $\alpha_{1}\left(t\right)\equiv \alpha_{2}\left(t\right)$. Below, we will show numerically that quantum synchronization can be reached in ${\hat{b}}_{1}$ and ${\hat{b}}_{2}$ for various settings.

**Figure 4:**
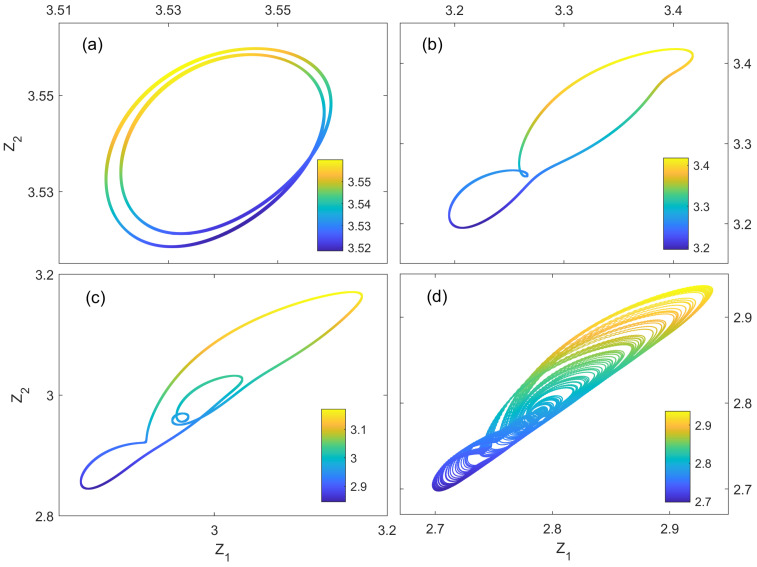
The phase-space orbits of the quantum mechanical resonator ${\hat{b}}_{1}$ for different classical optical detunings $\Delta_{c}/\Omega_{c}$: (**a**) −0.4, (**b**) −0.6, (**c**) −0.85, and (**d**) −0.95. Other parameters are set as follows: $\gamma_{c}/\Omega_{c}=1$, $g_{c}/\Omega_{c}=0.001$, $\Gamma_{c}/\Omega_{c}=0.001$, $\Omega_{c}/2\pi =1\;\mathrm{GHz}$, $\varepsilon_{c}/\Omega_{c}=418$, $\Delta_{1}/\Omega_{1}$ = −2, $\gamma_{1}/\Omega_{1}=1$, $\varepsilon_{1}/\Omega_{1}=0$, $g_{1}/\Omega_{1}=0.001$, $\Gamma_{1}/\Omega_{1}=10$, $\Omega_{1}/2\pi =0.01\;\mathrm{GHz}$, and T=0.002k. Here, $Z_{1}$ and $Z_{2}$ denote two coordinates of the 4-dimensional phase space ($Z_{1}$, $Z_{2}$, $Z_{3}$, and $Z_{4}$) reconstructed from the time series $\sigma_{1,x}\left(t\right)$ of the quantum mechanical resonator ${\hat{b}}_{1}$, and the 4-th coordinate $Z_{4}$ is scaled by colorbars shown in the figure.

In our simulations, we first prepare the classical controller ($\alpha_{c}$, $\beta_{c}$) to four different regimes by adjusting its detuning $\Delta_{c}/\Omega_{c}$: one-period (−0.4), two-period (−0.6), four-period (−0.85), and chaos (−0.95). Here, the initial conditions of two quantum mechanical modes are set as follows: $\sigma_{1,x}=\sqrt{1.5}$ and $\sigma_{2,x}=\sqrt{10.5}$. The corresponding phase space orbits of the quantum mechanical resonator ${\hat{b}}_{1}$ are shown in Figure 4; we find that the quantum dynamics are dominated by the classical controller ($\alpha_{c}$, $\beta_{c}$). Here, the phase portraits of the quantum system are reconstructed from the time-delayed coordinates $\sigma_{1,x}\left(\tau \right),\sigma_{1,x}\left(2\tau \right),\ldots ,\sigma_{1,x}\left(N\tau \right)$, where τ=0.3ns.

**Figure 5:**
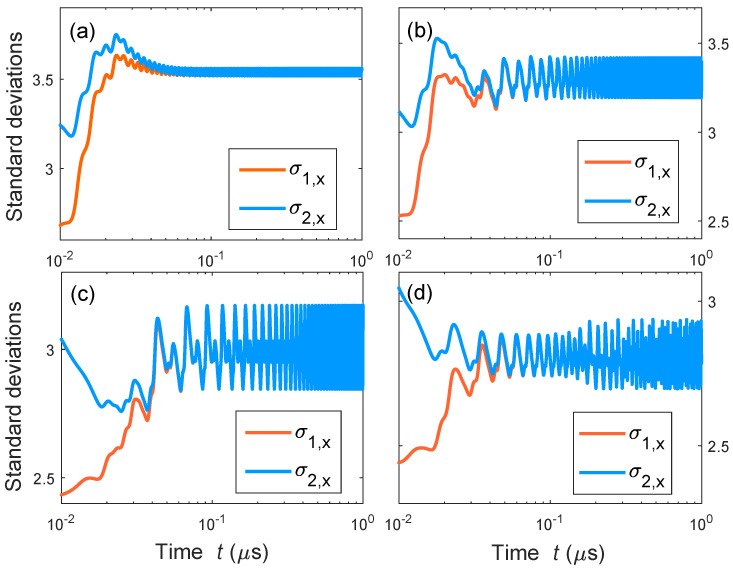
The standard deviations $\sigma_{1,x}$ and $\sigma_{2,x}$ of the quantum mechanical resonators ${\hat{b}}_{1}$ and ${\hat{b}}_{1}$ for different quantum motions given in Figure 4. Here, the parameters are set as follows: $\Delta_{1}/\Omega_{1}=\Delta_{2}/\Omega_{2}$ = −2, $\gamma_{1}/\Omega_{1}=\gamma_{2}/\Omega_{2}=1$, $\varepsilon_{1}/\Omega_{1}=\varepsilon_{2}/\Omega_{2}=0$, $g_{1}/\Omega_{1}=g_{2}/\Omega_{2}=0.001$, $\Gamma_{1}/\Omega_{1}=\Gamma_{2}/\Omega_{2}=10$, $\Omega_{1}/2\pi =\Omega_{2}/2\pi =0.01\;\mathrm{GHz}$. The other parameters are the same as in Figure 4.

The complete synchronization of two quantum mechanical resonators, ${\hat{b}}_{1}$ and ${\hat{b}}_{2}$, are presented in Figure 5. For each quantum regime given in Figure 4, the values of $\sigma_{1,x}\left(t\right)$ and $\sigma_{2,x}\left(t\right)$ merge together as the time increases, whereas they start from different initial conditions. The complete synchronization is shown to be robust for different quantum motions. The above numerical results are consistent with the analytic proof given in Section 3. The quantum synchronization is shown to be realizable in both stable and unstable regimes, e.g., quantum chaos in Figure 5d.

**Figure 6:**
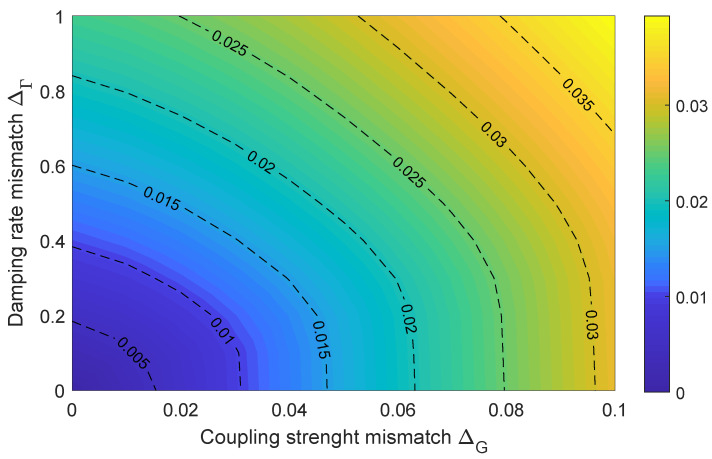
Average synchronization error $E_{\mathrm{avg}}$ for different mismatched parameters $\Delta_{\Gamma }$ and $\Delta_{G}$. Here, $\Delta_{c}/\Omega_{c}$ = −1, $g_{1}=10$ MHz, $\Gamma_{1}=0.15$ GHz, and $\Omega_{1}=\Omega_{2}=10$ MHz. The other parameters are the same as in Figure 4.

Moreover, we consider the cases in which two quantum mechanical modes, ${\hat{b}}_{1}$ and ${\hat{b}}_{2}$, have different parameters. To be more specific, here, we define the mismatched damping rate and mismatched resonant frequency as $\Delta_{\Gamma }=\left(\Gamma_{1}-\Gamma_{2}\right)/\Gamma_{1}$ and $\Delta_{G}=\left(G_{1}-G_{2}\right)/G_{1}$. Then, we introduce the average synchronization error, $E_{\mathrm{avg}}$, to measure the effect brought via these mismatched parameters: $E_{\mathrm{avg}}=∥\int_{t_{0}}^{\infty }e\left(t\right)dt/\int_{t_{0}}^{\infty }\sigma_{1,x}\left(t\right)dt∥$, where $t_{0}$ is the initial time. As shown in Figure 6, the values of $E_{\mathrm{avg}}$ are plotted in the Δ_Γ_-Δ_*G*_ plane and characterized by different colors. It can be seen that $E_{\mathrm{avg}}$ is less than 0.01, even when the mismatched damping rate, $\Delta_{\Gamma }$, is as high as 0.4; and $E_{\mathrm{avg}}$ is 0.03 when the mismatched coupling strength, $\Delta_{G}$, reaches the value 0.1. Here, it is shown that the complete synchronization of two quantum mechanical modes is still robust for the mismatched parameters $\Delta_{\Gamma }$ and $\Delta_{G}$.

**Figure 7:**
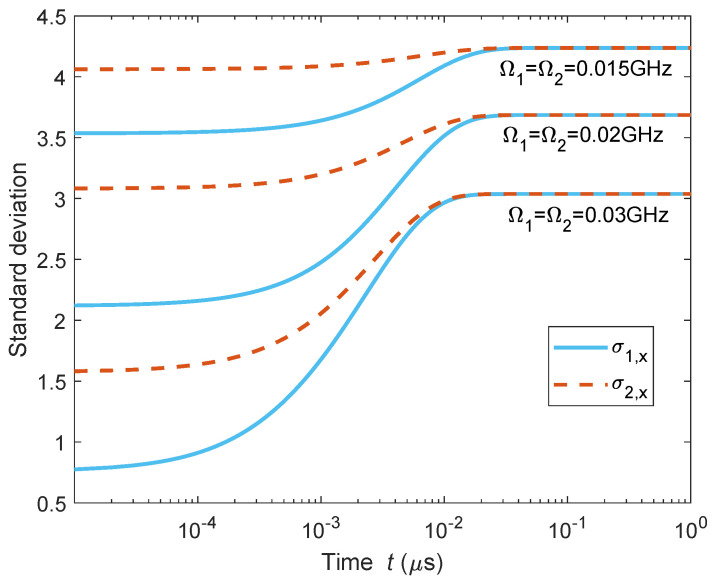
The standard deviations $\sigma_{1,x}$ and $\sigma_{2,x}$ of the quantum mechanical resonators ${\hat{b}}_{1}$ and ${\hat{b}}_{2}$ when the optomechanical couplings are absent ($g_{1}=g_{2}=0$). Here, the resonant frequencies ($\Omega_{1}$ and $\Omega_{2}$) of ${\hat{b}}_{1}$ and ${\hat{b}}_{2}$ are set to be three different values: (1) $\Omega_{1}=\Omega_{2}=0.015$ GHz, (2) $\Omega_{1}=\Omega_{2}=0.02$ GHz, and (3) $\Omega_{1}=\Omega_{2}=0.03$ GHz. The other parameters are the same as in Figure 4.

There is no doubt that the synchronization in this paper is caused by dissipation. Then, a natural question arises: Is the dissipation itself a sufficient condition for quantum synchronization? Or, in other words, is the APD model redundant for quantum synchronization? To answer this question, we consider the cases in which the two quantum harmonic oscillators are absent of common drivings but have the same dissipation. As shown in Figure 7, the standard deviations $\sigma_{1,x}$ and $\sigma_{2,x}$ of two quantum mechanical resonators, ${\hat{b}}_{1}$ and ${\hat{b}}_{2}$, merge together when they are not coupling to the optical modes ${\hat{a}}_{1}$ and ${\hat{a}}_{2}$ shown in Figure 3. However, it can also be seen that $\sigma_{1,x}$ and $\sigma_{2,x}$ stay at a constant value for each chosen resonant frequency. We prefer to call this a situation where two quantum systems stay at a non-oscillating stationary state instead of synchronization. Crucially, synchronization concerns the real-time relative relationship among interacting oscillators when the stationary cases are excluded. The APD configuration in this paper does not only drive quantum systems from stationary states; more importantly, it brings various motions, including chaos, into quantum systems, providing a feasible method for the general synchronizations of controllable quantum motions.

## 5. Conclusions and Discussion

In this paper, we have studied the synchronization of a continuous-variable system consisting of two quantum harmonic oscillators coupled with dissipative environments. We have shown that the active–passive decomposition configuration defined in classical dynamical systems plays a very important role in quantum regimes where quantum synchronization can be realized. For the physical models under consideration, it has been proven that quantum synchronization is asymptotically stable if quantum systems are subject to dissipation. Moreover, as an example, an experimentally accessible model based on an optomechanical setup was used to illustrate our approach to the quantum synchronization process defined in this paper. The numerical simulations clearly indicated that complete synchronization can be achieved and is robust with small parameter mismatches. It was shown that this quantum synchronization approach is robust with not only limit circles but also chaotic motions. The APD configuration we discussed in this paper is not limited to quantum harmonic oscillators; it can also be implemented with other quantum objects such as N-body qubits and atomic systems. Our future work will concentrate on constructing an APD model in synchronization quantum–classical hybrid systems, for which potential applications in quantum engineering, such as quantum computing, have been reported. It is desirable to consider quantum synchronization in different quantum open systems, such as dephasing noise, classical noises, and colored noise, which will be left to future publications.

## Data Availability

The data that support the findings of this study are available on request from the corresponding author.

## Abbreviations

The following abbreviations are used in this manuscript:

APD	Active–Passive Decomposition
